# An interpretable model for stock price movement prediction based on the hierarchical belief rule base

**DOI:** 10.1016/j.heliyon.2023.e16589

**Published:** 2023-05-26

**Authors:** Xiuxian Yin, Xin Zhang, Hongyu Li, Yujia Chen, Wei He

**Affiliations:** a Harbin Normal University, Harbin, 150025, China; b Rocket Force University of Engineering, Xi'an, 710025, China

**Keywords:** Stock price movement prediction, Interpretability, Belief rule base, Evidential reasoning

## Abstract

Stock price movement prediction is the basis for decision-making to maintain the stability and security of stock markets. It is important to generate predictions in an interpretable manner. The Belief Rule Base (BRB) has certain interpretability based on IF-THEN rule semantics. However, the interpretability of BRB in the whole process of stock prediction modeling may be weakened or lost. Therefore, this paper proposes an interpretable model for stock price movement prediction based on the hierarchical Belief Rule Base (HBRB-I). The interpretability of the model is considered, and several criteria are constructed based on the BRB expert system. First, the hierarchical structure of BRB is constructed to ensure the interpretability of the initial modeling. Second, the interpretability of the inference process is ensured by the Evidential Reasoning (ER) method as a transparent inference engine. Third, a new Projection Covariance Matrix Adaptive Evolution Strategy (P-CMA-ES) algorithm with interpretability criteria is designed to ensure the interpretability of the optimization process. The final mean squared error value of 1.69E-04 was obtained with similar accuracy to the initial BRB and enhanced in terms of interpretability. This paper is for short-term stock forecasting, and more data will be collected in the future to update the rules to enhance the forecasting capability of the rule base.

## 1 Introduction

The stock market is a crucial part of national economies and maintaining its stability and security is of great importance [1,2]. Due to the potential risks involved, analyzing the behavior and performance of stock markets has become a critical area of research [3]. One of the most essential tasks in this regard is predicting the movement of stock prices, as this information not only helps regulators stabilize financial markets but is also important for investors to make informed decisions and avoid risks. However, unreliable prediction results and unexplained prediction processes can lead to significant risks [4]. Hence, it is necessary to develop a reliable and convincing prediction model to mitigate potential risks.

In the current research, stock forecasting models are broadly classified into three categories: black-box models, white-box models, and gray-box models [[5], [6], [7]]. Black-box models, such as neural networks, are powerful in dealing with complex and nonlinear relationships, making them useful in many fields [8,9]. White-box models, such as linear regression, are simple and easy to interpret, allowing transparency and understanding of how the model arrives at its decisions [10,11]. While black-box models achieve high accuracy, they often lack interpretability, making it difficult for users to understand the reasons behind the predictions [12]. On the other hand, white-box models often sacrifice accuracy [13]. To address these issues, researchers are developing hybrid models that combine the advantages of both approaches. These models aim to provide accurate predictions that are also transparent and easy to understand, allowing users to make informed decisions based on the model's output [14,15].

The gray-box model combines the advantages of both black-box and white-box models to provide a balance between accuracy and interpretability [[14], [15], [16]]. It can capture complex relationships in the data like a black-box model, while also providing insight into the decision-making process like a white-box model. The model's architecture and parameters can be tuned to the domain knowledge of the problem, which enhances the accuracy and interpretability of the model. However, the development of such models remains an ongoing research topic and much work needs to be done to improve their accuracy and interpretability.

As a typical gray-box model, the BRB model is highly accurate because of its ability to handle uncertain and incomplete data [17,18]. It can integrate different sources of evidence to make accurate predictions and decisions. Second, BRB models allow for the incorporation of expert knowledge into the decision-making process [19]. This expert knowledge can help improve the accuracy and interpretability of the model, especially in cases where data are limited or incomplete. However, there are some problems with stock price prediction based on BRB. First, there are no detailed BRB interpretability criteria to ensure the availability of the model in the field of stock forecasting. Second, according to the proposed criteria, each part of the model needs to be adjusted to ensure the interpretability of the whole model.

In this study, a hierarchical BRB model is proposed which takes into account various interpretable criteria. The hierarchical structure is designed to avoid the problem of rule explosion problem, which limits the application of BRB to multi-attribute systems. The interpretable criteria are referred to the BRB interpretable general criteria proposed by Cao et al. [5] and are intended to be applied for the maintenance of the interpretability of stock forecasts. HBRB-I is expected to provide accurate and interpretable forecasts for stock markets.

The main contributions are as follows:

a) To ensure the interpretability of the predictive model, several criteria were developed based on the BRB expert system. These criteria include model structure, input-output inference relationships, and parameter optimization processes. The criteria proposed in this paper can guide building BRB-based prediction models.

b) A specific process for initializing, inferring, and optimizing the interpretable prediction model is developed based on the interpretability criteria. The model is initialized using a hierarchical structure with an initial rule base and parameters, and then the inference process is performed using a transparent inference algorithm. Finally, the parameters are optimized using an adapted optimization model. The proposed hierarchical structure with an improved interpretable optimization algorithm solves the problem of rule explosion and corrupted interpretability of BRB.

The rest of this paper is organized as follows. In Section 2, past research is reviewed. In Section 3, the problems faced by BRB for stock price movement prediction are summarized. In Section 4, the interpretability criteria of the stock price movement prediction method are proposed. In Section 5, the HBRB-I model is constructed based on the interpretability criteria. In Section 6, a case study is conducted. In Section 7, the paper is summarized.

## 2 Literature review

Stock price movement forecasting is a time-varying forecasting task and it is crucial to study the relevant time series forecasting methods.

Various time series forecasting techniques used in stock price movement forecasting include autoregressive integrated moving average (ARIMA), exponential smoothing (ETS), and seasonal decomposition of time series (STL), among others. Khan et al. compared three-time series forecasting models for accurate stock market forecasting [20]. The study uses historical data for Netflix stock over five years and compares the performance of automated ARIMA as well as two customized ARIMA models. The results show the potential of using ARIMA models in stock market forecasting, especially when combined with historical data. Sun et al. applied two popular methods, the autoregressive integrated moving average (ARIMA) and exponential smoothing (ETS), to predict the closing stock market prices of individual stocks [21]. Standard deviation is used for evaluation. The study concludes that the ARIMA model performs better than ETS and shows promising general trend forecasts compared to existing methods. He et al. proposed a new approach to financial time series forecasting by using STL and dendritic neuron models [22]. The model outperforms existing models on 16 real-world stock market indices, allowing for a better understanding of financial data and improved prediction accuracy.

According to the model mechanism, it can be further divided into the following three types. In the table, the relevant types of literature are described, as well as their advantages and disadvantages.

**Table:** 

Model	Advantages and disadvantages	Current status of research
**Black-box models**	Black-box models based on observation data are favored in the field of stock price prediction due to their good operability and modeling accuracy. However, the accuracy of black-box models depends on data samples, the modeling process is not interpretable, and the internal parameters and structure are difficult to understand [23,24].	Tsantekidis et al. used a convolutional neural network (CNN)-based stock prediction model and compared it with other classical models to verify the effectiveness of convolutional models in stock prediction [25]. Gen et al. demonstrated that ANN-based nonlinear models outperformed linear prediction models for stock market prediction problems [26]. Mohammad et al. combined the ARIMA model, BP neural network, and multilayer perceptron (MLP) models for stock prediction research, and the hybrid model achieved better prediction results [27].
**White-box models**	White-box models do not depend on the observed data and provide a transparent modeling process and interpretable results. However, the accuracy of the white-box model is limited due to the harsh operating environment and the complex model structure [28].	Hindrayani et al. observed the data stocks of telecommunication companies and generalized the inference for each prediction decision for each company stock by constructing a decision tree model, and the final results obtained the smallest mean absolute percentage error [29]. Shakeri et al. proposed an expert system based on fuzzy rules. Its applicability to the daily transactions of speculators and traders in the stock market despite the uncertainty and ambiguity of the environmental parameters [30].
**Gray-box models**	Gray-box models construct the model by model mechanism and optimize the model by using data samples, which guarantees the accuracy of the model [5,14]. However, the gray-box model requires a certain level of expertise in both data science and domain knowledge, which can be a disadvantage for users with limited expertise in either field [31].	Gao et al. first applied the Evidential Reasoning (ER) rule to synthesize the opinions of financial analysts for stock investment decisions. It is proven that ER rule is effective for making financial investment decisions [32]. Hossain et al. used the technical analysis of the belief rule base (BRB) expert system combined with the Bollinger band to predict stock prices for the next five days. The final results show the potential of BRB in predicting the price movements of financial assets [33].

BRB has been widely used in various fields due to its good performance in gray-box modeling. The following table briefly describes the current status of BRB development in the interpretable direction.

**Table:** 

BRB model with interpretability	Brief description
Yang et al. proposed a belief rule base inference method based on an evidence-based inference algorithm in 2006 [34].	This method introduces a confidence framework to traditional generative rules based on the Dempster-Shafer evidence theory, decision theory, fuzzy theory, and traditional generative rules. It can provide a more reliable description of knowledge in engineering.
A unified guide to interpretable BRB expert systems was established by Cao et al. [5].	The article systematically summarizes the interpretable features of BRBs and provides guidelines for the establishment of interpretable BRBs in the future.
Zhou et al. proposed a health state assessment model with interpretable BRB [35].	The model considers the interpretable BRB modeling criterion and proposes three concepts to maintain the interpretability of the optimization process.
Han et al. proposed an interpretable BRB model for lithium battery capacity prediction [7].	The model proposes a new interval optimization strategy that ensures a balance between accuracy and interpretability.

## 3 Problem formulation

For the two problems of the HBRB-I prediction method, the accuracy and interpretability of the model are considered, and a prediction model of hierarchical BRB with interpretability is established. The specific problems are as follows.

**Problem Ⅰ:** How to go about summarizing the BRB interpretability criterion applicable to prediction models based on the BRB interpretability general criteria proposed by previous researchers. Cao et al. performed a comprehensive review of BRB interpretability and put forward eight general criteria $\left\{C_{general}|C_{1},C_{2},...,C_{8}\right\}$ to guide the establishment of interpretable BRBs [5]. These criteria can serve as a reference for future BRB research. It is important to ensure that the entire modeling process is as interpretable as possible. Therefore, this paper proposes interpretable criteria based on general criteria as Eq. (1):

(1) $$\text{Interpretability}\;\text{criteria}:\left\{C|C_{1},C_{2},...,C_{n}\right\}\text{,}$$

where *C* denotes the interpretable criteria set, and *n* denotes the criteria number.

**Problem Ⅱ:** How to build interpretable prediction models based on the interpretability criteria. According to the proposed interpretability criteria for stock price prediction, it is necessary to adjust each part of the model [36]. In building the model, reasoning, and optimization, the computational soundness and the cause-effect relationship between inputs and outputs must be fully considered [7].

The first question is how to construct a suitable model structure, described as Eq. (2):

(2) $$\psi =\vartheta \left[X_{1},X_{2},\ldots ,X_{m}\right]$$

where *X*_*j*_*(j=1,2, …,m)* represents the prior attribute input of the system. ψ represents the constructed rational model structure. ϑ represents the construction process.

The next problem is how to improve the optimization algorithm, described in Eq. (3).

(3) $$\Omega_{best}=\Xi \left(\Omega ,\varpi \right)$$

where Ω represents the set of parameters of the optimization process. ϖ represents the interpretable constraints set by the expert. Ξ represents the optimization process of the parameters. $\Omega_{best}$ represents the optimal parameters after optimization.

The final model inference is described as Eq. (4):

(4) y=f(x,C,EK)

where *x* denotes the input data of the stock prediction system. *EK* represents expert knowledge, which is used for the setting of rule base parameters Ω and interpretability parameters for ϖ. *y* denotes the set of stock price movement prediction results. *f* denotes the nonlinear function to represent the relationship between the system characteristics and the prediction value.

### 3.1 Interpretability of BRB for stock price movement prediction

**Fig. 1:**
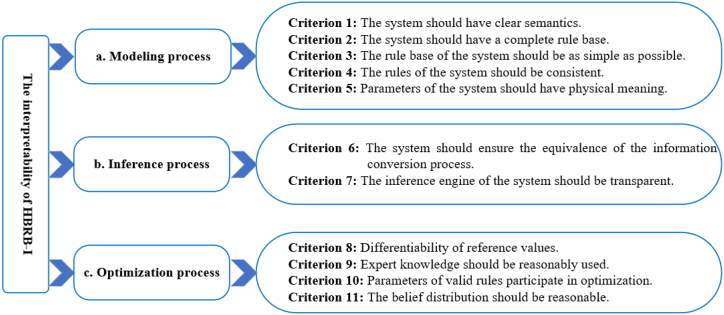
The interpretability of HBRB-I.

The stock market has very high requirements for the interpretability of models. Some popular data-driven models are not able to meet the requirements of the stock market. Although the initial BRB expert system has the advantage of interpretability [37], it still cannot guarantee its global interpretability in the stock market. Therefore, based on the general criterion of interpretability of BRB proposed by Cao et al. [5], several interpretability criteria are developed for stock price movement prediction. Considering the global interpretability of BRB should be specific in three aspects: model construction, inference, and optimization from Fig. 1.

### 3.2 Modeling process

**Criterion 1:** The system should have clear semantics.

First, the reference values of IF-THEN rule inputs and their matching intervals possess semantic distinguishability to represent clear semantics [[37], [38], [39]]. Second, the standardization of the matching degree can form an easily understandable semantic. Match normalization ensures that every reference value has at least one data point in the domain *X* with a match score of 1, and all match scores are between 0 and 1 [37,39], which can be described as Eq. (5):

(5) $$\begin{array}{l} \forall 1\leq \upsilon \leq T,\exists x_{p}\in X,a_{\upsilon }\left(x_{p}\right)=1\text{,}\\ \forall 1\leq \upsilon \leq T,x\in X,0\leq a_{\upsilon }\left(x\right)\leq 1\text{,} \end{array}$$

where *T* denotes the number of reference values of the predicate attribute, $x_{p}$ denotes a certain fixed value in the domain. $a_{\upsilon }\left(x\right)$ denotes the degree of matching concerning the υ th reference value and *X* denotes the entire feasible domain of *x*.

**Criterion 2:** The system should have a complete rule base.

Completeness of the rule base means that at least one reference value should be matched for any possible input, and at least one rule should be activated, which can be described as Eq. (6). In other words, it is understandable that all working states should be included in the rule base [40].

(6) $$\forall x\in X\left\{ \begin{array}{l} \exists 1\leq \upsilon \leq T,a_{\upsilon }\left(x\right)>0\\ \exists 1\leq l\leq L,0<w_{l}\leq 1, \end{array}\right.$$

where *L* denotes the number of rules and $w_{l}$ denotes the activation weight of the *l*th rule.

In BRB, a “working state” is a possible combination of input variables that can trigger one or more rules in a rule base [34]. For example, if a rule base has two input variables, A and B, and each variable has two possible states, “high” or “low”, then there are a total of four possible working states, {(A, high), (B, high)},{(A, high), (B, low)}, {(A, low), (B, high)}, {(A, low), (B, low)}.

**Criterion 3:** The rule base of the system should be as simple as possible.

The simple rule base is the key part of BRB interpretability [41], which is beneficial for researchers to understand the global system easily and obtain higher model performance [5,42]. For BRB, a simple rule base is a set of rules that is concise, easy to understand, and has a small number of antecedents and consequences [43]. To evaluate whether a given rule base is simple, we can consider the number of rules as well as the number of antecedent and consequent parameters. A simple rule base will usually have fewer rules and a smaller number of antecedents and consequences [42]. This criterion is also a research hotspot for researchers. The simplicity of the rule base greatly limits the application of BRB. At present, it is a very suitable method to construct a reasonable structure or conduct feature screening [34]. The following Eq. (7) shows the size of the rule base for computing a certain 4-attribute system, it is clear that this makes the size of the rule base much smaller.

(7) $$\left\{ \begin{array}{l} N_{A}=T_{\delta_{a}}*T_{\delta_{b}}*T_{\delta_{c}}*T_{\delta_{d}}\text{,}\\ N_{B}=T_{\delta_{a}}*T_{\delta_{b}}+T_{\delta_{c}}*T_{\delta_{d}}+T_{\delta_{a,b}}*T_{\delta_{c,d}}\text{,} \end{array}\right.$$

where $N_{A}$ and $N_{B}$ denote the size of the directly constructed and hierarchically constructed rule bases, respectively. $T_{\delta_{i}}\left(i=a,b,c,d\right)$ indicate the number of reference values for the prior attribute. $T_{\delta_{a,b}}$ and $T_{\delta_{c,d}}$ indicate the number of next-level attribute parameters.

**Criterion 4:** The rules of the system should be consistent.

The consistency of rules can effectively prevent the ambiguity of the final result. Conflicting rules cannot be understood and are not allowed to exist in the modeling process [5,34]. It is a good method to extract expert knowledge and transform it into rules and construct a rule base.

**Criterion 5:** Parameters of the system should have physical meaning.

The parameters with physical meaning are the basis of the interpretable model. If the parameters have no meaning, the whole method is meaningless. The parameters of the BRB model mainly include belief degree, rule weight, attribute weight, and activation weight, which have their physical significance. They are all between 0 and 1, which can be described as Eq. (8):

(8) {δ,θ,β,w}∈[0,1],

where β denotes the belief degree. θ denotes the rule weight. δ denotes the feature weight. w denotes the activation weight.

### 3.3 Inference process

**Criterion 6:** The system should ensure the equivalence of the information conversion process.

In the process of inference, the system should try to maintain the integrity of the initial information and have reasonable information conversion in the belief structure. The ER method based on rule and utility is a better algorithm, which has the equivalent and reasonable information conversion ability in the belief structure [44,45].

**Criterion 7:** The inference engine of the system should be transparent.

BRB is a popular and effective method for decision-making in various domains, but it is necessary to ensure that the inference algorithm maintains the interpretability of the rule base and provides a transparent inference process to obtain understandable results. In this regard, the ER method is a transparent reasoning method that can effectively guarantee the interpretability of the model inference process [23,45,46].

### 3.4 Optimization process

**Criterion 8:** Differentiability of reference values.

The initial reference value and the best reference value should be in the feasible region preliminarily judged by experts, which can be described as Eq. (9):

(9) $$\begin{array}{l} {\left(\beta ,\theta ,\delta \right)}_{low}\leq {\left(\beta ,\theta ,\delta \right)}_{initial}\leq {\left(\beta ,\theta ,\delta \right)}_{up}\text{,}\\ {\left(\beta ,\theta ,\delta \right)}_{low}\leq {\left(\beta ,\theta ,\delta \right)}_{optimal}\leq {\left(\beta ,\theta ,\delta \right)}_{up}\text{,} \end{array}$$

(β,θ,δ)_*initial*_ and (β,θ,δ)_*optimal*_ denote the initial expert knowledge and optimized expert knowledge, respectively. (β,θ,δ)_*low*_ and (β,θ,δ)_*up*_ denote the space of the feasible region.

**Criterion 9:** Expert knowledge should be reasonably used.

Expert knowledge is an important part of interpretability, and the optimization process should be based on expert judgment for local search [47]. Therefore, expert knowledge is introduced into the initial population [48], and Euclidean distance is introduced to further realize local search domain optimization [6], which can be described as Eqs. (10), (11):

(10) $$m^{\left(g\right)}=\left\{ \begin{array}{l} EK,if\;g=1\\ m^{\left(g\right)},if\;g\neq 1 \end{array}\right.$$

where $m^{\left(g\right)}$ denotes the *g*th generation population.

(11) $$\rho \left(x_{n},x_{n}^{\prime }\right)=\sqrt{\sum_{i=1}^{n}{\left(x_{i}-x_{i}^{\prime }\right)}^{2}}\leq d$$

$\rho \left(x_{n},x_{n}^{\prime }\right)$ is the Euclidean distance between the initial population of individuals and the expert knowledge. *d* is the distance parameter determined by experts.

**Criterion 10:** Parameters of valid rules participate in optimization.

Assume that the parameter vector of the BRB is represented as Eq. (12):

(12) Ω=(β,θ,δ),

Ιf *i*th rule is activated, then the relevant activated parameter $\left(\beta_{1}^{i},\beta_{2}^{i},...,\beta_{N}^{i},\theta_{i},\delta_{1},\delta_{2},...,\delta_{T}\right)$ can participate in the optimization, while other unactivated parameters should continue to maintain the initial expert knowledge. Therefore, it is necessary to distinguish inactive rules for effective work, which can be expressed as Eqs. (13), (14):

(13) $$\omega_{k}=\left\{ \begin{array}{l} 0,W_{k}=0,\\ 1,otherwise, \end{array}\right.$$

(14) $$W_{k}=\left(w_{1},w_{2},...,w_{P}\right),k=1,2,...,L$$

where ω is used to discriminate the unactivated rules, the size of the dataset is *P*, and *W*_*k*_ is the activation weight vector computed from the dataset. If the parameters of the unactivated rules are optimized, the correction of the initial expert knowledge is performed as Eq. (15):

(15) $$\Omega_{m}^{\left(g+1\right)}⇐BRB_{initial}\left(\beta_{k},\theta_{k}\right)\text{,}$$

where $\Omega_{m}^{\left(g+1\right)}$ denotes the *m*th parameter vector. $BRB_{initial}\left(\beta_{k},\theta_{k}\right)$ denotes the parameter associated with the *k*th rule in the initial expert knowledge base. ⇐ is the replacement operation, which replaces the overoptimized parameter, thereby forming a new $\Omega_{m}^{\left(g+1\right)}$ that meets the interpretability.

**Criterion 11:** The belief distribution should be reasonable.

**Fig. 2:**
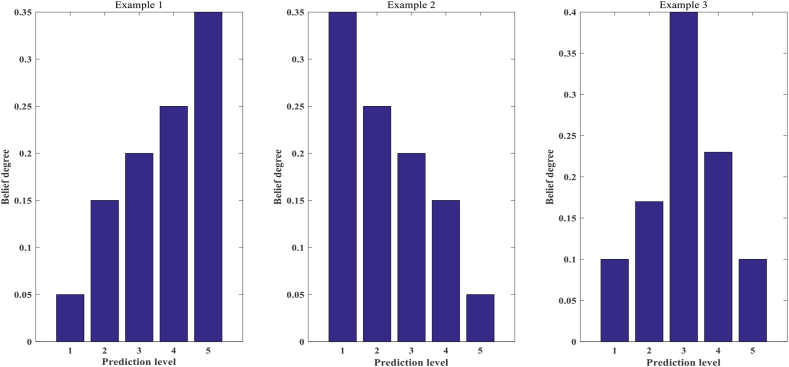
Examples of the right belief distribution.

There are three evaluation levels for students: excellent, good and poor. The teacher's evaluation is {(excellent, 0.8), (good, 0.2), (poor, 0)}. However, the confidence distribution after optimization may be {(excellent, 0.6), (good, 0), (poor, 0.4)}, this is inexplicable. As shown in Fig. 2, a reasonable shape of the belief distribution in this system should be monotonic or convex. For example, the interpretable criterion of the *k*th rule can be expressed as Eqs. (16), (17):

(16) $$\beta_{i}^{k}∼C_{11},i=1,2,...,N$$

(17) $$\begin{array}{c} C_{11}=\{\left(\beta_{1}^{k}\leq \beta_{2}^{k}\leq \cdots \leq \beta_{N}^{k}\right)\vee \left(\beta_{1}^{k}\geq \beta_{2}^{k}\geq \cdots \geq \beta_{N}^{k}\right)\\ \vee \left(\beta_{1}^{k}\leq \cdots \leq \max\left(\beta_{2,...,N-1}^{k}\right)\geq \cdots \geq \beta_{N}^{k}\right)\} \end{array}$$

$\beta_{i}^{k}\left(i=1,2,...,N\right)$ is the *k*th belief distribution that satisfies the interpretability criterion $C_{11}$.

## 4 The stock price movement prediction method based on the HBRB-I

HBRB-I is constructed based on a prediction study of stock price movements in subsection 5.1. The process of rule inference using the ER is described in subsection 5.2. The optimization process is implemented in subsection 5.3. These subsections are based on the criteria in Section 4.

### 4.1 Model construction

BRB is a rule base using the evidential reasoning method proposed by Yang et al. [34]. In the method, the *k*th IF-THEN belief rule is expressed as Eq. (18):

(18) $$\begin{array}{c} R_{k}:\text{IF}\left(X_{1}\;is\;A_{1}^{k}\right)\Lambda \left(X_{2}\;is\;A_{2}^{k}\right)\Lambda \cdots \Lambda \left(X_{T_{k}}\;is\;A_{T_{k}}^{k}\right)\text{,}\\ \text{THEN}\left\{\left(D_{1},\beta_{1}^{k}\right),\left(D_{2},\beta_{2}^{k}\right),\ldots ,\left(D_{N},\beta_{N}^{k}\right)\right\},\left(\sum_{i=1}^{N}\beta_{i}^{k}\leq 1\right)\\ \text{with}\;a\;\text{rule}\;\text{weight}\;\theta_{k}\left(k=1,2,\ldots ,L\right)\\ \text{and}\;\text{attribute}\;\text{weights}\;\delta_{i}\left(i=1,2,\ldots ,T_{k}\right)\text{,} \end{array}$$

where $X_{1},X_{2},\ldots ,X_{T_{K}}$ are the antecedent attributes of the stock price movement prediction method. $A_{i}^{k}\left(i=1,2,\ldots ,T_{k}\right)$ denotes the referential value. $\theta_{k}$ is the weight of the *k*th rule. $\delta_{i}\left(i=1,2,\ldots ,T_{k}\right)$ denotes the weight of the *i*th feature. *L* denotes the number of rules and $T_{k}$ is the number of antecedent attributes. *D*_*N*_ denotes the prediction result and $\beta_{i}^{k}\left(i=1,2,\ldots ,N\right)$ represents the belief degree.

Because the rule base of the BRB is created by IF-THEN rules, the structure of the model can be understood more clearly. Here is an example, as described in Eq. (19):

(19) $$\begin{array}{c} \text{IF}\left(High\;price\;is\;high\right)\Lambda \left(Low\;price\;is\;high\right)\text{,}\\ \text{THEN}\left\{\left(High,0.9\right),\left(Midum,0.1\right),\left(Low,0\right)\right\}\\ \text{with}\;\text{rule}\;\text{weight}\;\theta =1\\ \text{and}\;\text{attribute}\;\text{weights}\;\delta_{1}=1,\delta_{2}=1 \end{array}$$

It can be seen that BRB can handle qualitative and quantitative information well, and the modeling is easy to understand. However, in the application of BRB, due to a large number of influential indicators, too many input attributes can lead to rule explosion. Therefore, considering the multiple attributes of the stock index, the hierarchical structure of the model is designed, which makes the structure of the model have good scalability and can be better applied to the stock price movement prediction problem.

**Fig. 3:**
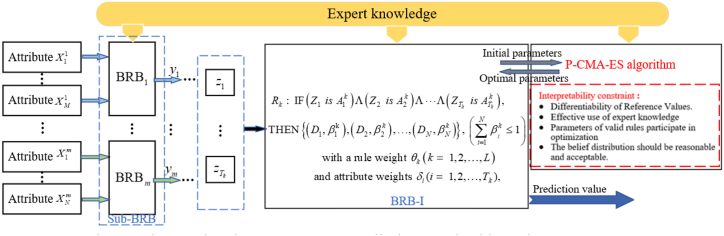
The stock price movement prediction method based on HBRB-I.

All attributes within the method are divided into groups according to their attribute characteristics and the corresponding result sets are introduced based on the BRB established by expert knowledge, and then continued as input values for the next layer until the last layer [34,49]. Then use the optimization algorithm considering the interpretability criterion to optimize. Ultimately, high-accuracy prediction results are derived under the interpretable model. The method structure is shown in Fig. 3.

### 4.2 Evidential reasoning method

First, before the inference process begins, the degree of match for each reference value is determined to generate the activation weights of rules. Then, ER method merges rules and generates conclusions [34].

Then, the specific inference process is shown below:

**Step 1.** The antecedent attributes of the BRB are derived from the system characteristics. First, observed data of system properties are transformed into belief distributions, which can be described as Eqs. (20), (21):

(20) $$S\left(x_{i}\right)=\left\{\left(A_{i,j},a_{i,j}\right),i=1,2,...,M;j=1,2,...,J_{i}\right\}\text{.}$$

(21) $$\begin{array}{l} a_{i,j}=\frac{A_{i,j+1}-x_{i}}{A_{i,j+1}-A_{i,j}},a_{i,j+1}=1-a_{i,j},A_{i,j}\leq x_{i}\leq A_{i,j+1}\\ a_{i,l}=0,for\;l=1,2,...,L\&l\neq j\&l\neq j+1 \end{array}$$

where *a*_*i,j*_ denotes the matching degree. *M* denotes the number of data and *L* denotes the number of parameters of the attribute.

**Step 2.** The rules in the BRB are activated in different degrees based on the observation data inputs of system characteristics, and their activation weights can be determined by Eqs. (22), (23):

(22) $$w_{k}=\frac{\theta_{k}\prod_{i=1}^{T}{\left(\alpha_{i,j}^{k}\right)}^{{\bar{\delta }}_{i}}}{\sum_{l=1}^{L}\theta_{l}\prod_{i=1}^{T}{\left(\alpha_{i,j}^{k}\right)}^{{\bar{\delta }}_{i}}}\text{,}$$

(23) $${\bar{\delta }}_{i}=\delta_{i}/\max_{i=1,2,...T_{k}}\left\{\delta_{i}\right\}\text{,}$$

where ${\bar{\delta }}_{i}$ denotes the normalized weight of the *i*th attribute. $w_{k}$ denotes the activation weights for the kth rule.

**Step 3.** The activated rules generate their output belief degrees, and the evidential reasoning algorithm is employed to integrate the activated rules. The final output belief degree is then calculated by Eq. (24):

(24) $$\beta_{n}=\frac{\left[\prod_{k=1}^{L}\left(w_{k}\beta_{n}^{k}+1-w_{k}\sum_{i=1}^{N}\beta_{i}^{k}\right)-\prod_{k=1}^{L}\left(1-w_{k}\sum_{i=1}^{N}\beta_{i}^{k}\right)\right]}{\left[\sum_{n=1}^{N}\prod_{k=1}^{L}\left(w_{k}\beta_{n}^{k}+1-w_{k}\sum_{i=1}^{N}\beta_{i}^{k}\right)-\left(N-1\right)\prod_{k=1}^{L}\left(1-w_{k}\sum_{i=1}^{N}\beta_{i}^{k}\right)\right]-\left[\prod_{k=1}^{L}\left(1-w_{k}\right)\right]}$$

**Step 4.** The final output belief distribution is then indicated by Eq. (25):

(25) $$S\left(A^{•}\right)=\left\{\left(D_{n},\beta_{n}\right);n=1,2,...,N\right\}\text{,}$$

where $A^{•}$ denotes the input data.

**Step 5.** The final predictive value can be obtained by using the calculated output belief degree by Eq. (26):

(26) $$u\left(S\left(A^{•}\right)\right)=\sum_{n=1}^{N}u\left(D_{n}\right)\beta_{n}\text{,}$$

where *u(D*_*n*_*)* denotes the utility of *D*_*n*_. $u\left(S\left(A^{•}\right)\right)$ denotes the expected utility.

### 4.3 Model optimization process

**Fig. 4:**
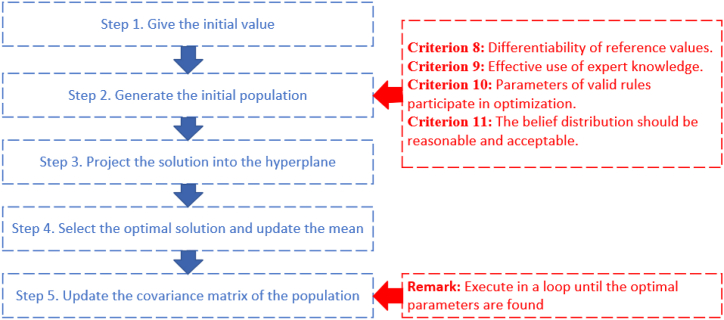
P-CMA-ES optimization procedure.

The projection covariance matrix adaptation evolution strategy (P-CMA-ES) can be well applied to the optimization problem of BRB [5,50]. The algorithm is a relatively advanced algorithm used to solve complex nonlinear and discontinuous convex optimization problems, and it was first proposed by Hansen [51]. The brief steps of the algorithm are shown in Fig. 4.

First, the objective function under the new experiment is constructed by Eq. (27):

(27) $$\begin{array}{l} \text{Min}\left\{MSE\left(\beta ,\theta ,\delta \right)\right\}\\ s.t.\;{\left(\beta ,\theta ,\delta \right)}_{low}\leq {\left(\beta ,\theta ,\delta \right)}_{initial}\leq {\left(\beta ,\theta ,\delta \right)}_{up}\text{,}\\ {\left(\beta ,\theta ,\delta \right)}_{low}\leq {\left(\beta ,\theta ,\delta \right)}_{optimal}\leq {\left(\beta ,\theta ,\delta \right)}_{up}\text{,}\\ \sum_{i=1}^{N}\beta_{i}^{k}\leq 1,\beta_{i}^{k}∼C_{11}\left(i=1,2,...,N\right) \end{array}$$

where MSE(*) denotes the degree of comprehensive evaluation.

**Step 1.** Give the initial values of the following variables:

Population size λ, offspring population size μ, covariance matrix $C^{0}$, population $m^{0}=\Omega^{0}\left(\beta ,\theta ,\delta \right)$ and step size $\varepsilon^{0}$.

**Step 2.** Generate the population by Eq. (28):

(28) $$\Omega_{k}^{g+1}=m^{\left(g\right)}+\varepsilon^{\left(g\right)}N\left(0,C^{\left(g\right)}\right),k=1,2,...,\lambda$$

where *N* denotes the normal distribution.

Interpretability Criterion 9, which can be described as Eqs. (29), (30):

(29) $$m^{\left(g\right)}=\left\{ \begin{array}{l} EK,if\;g=1\\ m^{\left(g\right)},if\;g\neq 1 \end{array}\right.$$

(30) $$\rho \left(x_{n},x_{n}^{\prime }\right)=\sqrt{\sum_{i=1}^{n}{\left(x_{i}-x_{i}^{\prime }\right)}^{2}}\leq d$$

Interpretability criterion 9 ensures the initial interpretability by converting expert knowledge into parameters and slightly adjusting parameters.

Interpretability Criterion 10, which can be described as Eq. (31):

(31) $$\Omega_{m}^{\left(g+1\right)}⇐BRB_{initial}\left(\beta_{k},\theta_{k}\right)$$

All unactivated parameters are replaced in the optimization process to form the final parameters according to the marked unactivated rules. The interpretability is fully ensured, and the trust of the researcher in the method is increased.

Interpretability Criterion 8, which can be described as Eq. (32):

(32) $$C_{8}=\left\{ \begin{array}{l} \theta_{lp_{k}}\leq \theta_{k}\leq \theta_{up_{k}}k\in \left\{1,2,...,L\right\}.\\ \delta_{lp_{j}}\leq \delta_{j}\leq \delta_{up_{j}}j\in \left\{1,2,...,T_{k}\right\}.\\ \beta_{n_{lp_{k}}}\leq \beta_{n}^{k}\leq \beta_{n_{up_{k}}}\;n\in \left\{1,2,...,N\right\}. \end{array}\right.$$

The optimization range is carried out within the feasible interval given by the experts, and the criteria are added to the initial judgment of the experts to achieve.

Interpretability Criterion 11, which can be described as Eqs. (33), (34):

(33) $$\beta_{k}^{\left(g+1\right)}∼C_{11},k=1,2,...,\lambda$$

(34) $$\begin{array}{c} C_{11}=\{\left(\beta_{1}^{k}\leq \beta_{2}^{k}\leq \cdots \leq \beta_{N}^{k}\right)\vee \left(\beta_{1}^{k}\geq \beta_{2}^{k}\geq \cdots \geq \beta_{N}^{k}\right)\\ \vee \left(\beta_{1}^{k}\leq \cdots \leq \max\left(\beta_{2,...,N-1}^{k}\right)\geq \cdots \geq \beta_{N}^{k}\right)\} \end{array}$$

The interpretability criterion 11 adjusts the rules that deviate from the correct semantic distribution through the interpretability criterion.

**Step 3.** Project the solution into the hyperplane by Eq. (35):

(35) $$A_{e}\Omega_{k}^{\left(g+1\right)}\left(1+n_{e}\left(j-1\right):n_{e}j\right)=1,j=1,2,...,N+1$$

where *A*_*e*_ is the parameter set. *n*_*e*_ and *j* denote the quantities of variables in equality constraints and equality constraints in $\Omega_{k}^{\left(g\right)}$ respectively.

The projection operation is described in Eq. (36):

(36) $$\Omega_{k}^{\left(g+1\right)}\left(1+n_{e}\left(j-1\right):n_{e}j\right)=\Omega_{k}^{\left(g+1\right)}\left(1+n_{e}\left(j-1\right):n_{e}j\right)-A_{e}^{T}{\left(A_{e}A_{e}^{T}\right)}^{-1}\times \Omega_{k}^{\left(g+1\right)}\left(1+n_{e}\left(j-1\right):n_{e}j\right)A_{e}\text{.}$$

**Step 4.** Select the optimal solution and update the mean:

Calculate and sort the MSE value by Eq. (37):

(37) $$MSE\left(\Omega_{1:\lambda }^{\left(g+1\right)}\right)\leq MSE\left(\Omega_{2:\lambda }^{\left(g+1\right)}\right)\leq \cdots \leq MSE\left(\Omega_{\lambda :\lambda }^{\left(g+1\right)}\right)\text{,}$$

where $\Omega_{a:\lambda }^{\left(g+1\right)}$ is the *a*th solution in λ solutions.

The subgroup is calculated by Eq. (38):

(38) $$m^{\left(g+1\right)}=\sum_{i=1}^{\mu }\varsigma_{i}\Omega_{i:\lambda }^{\left(g+1\right)}\text{.}$$

**Step 5.** Update the covariance matrix of the population by Eqs. (39), (40):

(39) $$C^{\left(g+1\right)}=\left(1-c_{1}-c_{2}\right)C^{\left(g\right)}+c_{1}p_{c}^{\left(g+1\right)}{\left(p_{c}^{\left(g+1\right)}\right)}^{T}+c_{2}\sum_{i=1}^{\mu }\varsigma_{i}\left(\frac{\Omega_{i:\lambda }^{\left(g+1\right)}-m^{\left(g\right)}}{\varepsilon^{\left(g\right)}}\right){\left(\frac{\Omega_{i:\lambda }^{\left(g+1\right)}-m^{\left(g\right)}}{\varepsilon^{\left(g\right)}}\right)}^{T}$$

(40) $$p_{c}^{\left(g+1\right)}=\left(1-c_{c}\right)p_{c}^{\left(g\right)}+\sqrt{c_{c}\left(2-c_{c}\right){\left(\sum_{i=1}^{\mu }\varsigma_{i}^{2}\right)}^{-1}}\left(m^{\left(g+1\right)}-m^{\left(g\right)}\right)/\varepsilon^{\left(g\right)}$$

where the step size $\varepsilon^{g+1}$ is updated by Eqs. (41), (42):

(41) $$\varepsilon^{g+1}=\varepsilon^{g}\;\exp\left(\frac{c_{\sigma }}{d_{\sigma }}\left(\frac{∥p_{\sigma }^{\left(g+1\right)}∥}{E∥N\left(0,1\right)∥}-1\right)\right)$$

(42) $$\begin{array}{l} p_{\sigma }^{\left(g+1\right)}=\left(1-c_{c}\right)p_{\sigma }^{\left(g\right)}+\sqrt{c_{c}\left(2-c_{c}\right){\left(\sum_{i=1}^{\mu }\varsigma_{i}^{2}\right)}^{-1}}\\ {\left(C^{\left(g\right)}\right)}^{-\frac{1}{2}}\left(m^{\left(g+1\right)}-m^{\left(g\right)}\right)/\varepsilon^{\left(g\right)} \end{array}$$

where *c*_*1*_ and *c*_*2*_ denote the learning rate, and *p*_*c*_ denotes the evolution path. *c*_*c*_ is the parameter of the evolution path. $d_{\sigma }$ denotes the damping parameter.

## 5 Case study

The experimental procedure is described in Section 6.1. The dataset and experimental setup are described in subsection 6.2. The experimental procedure is described in subsection 6.3, and the final analysis is discussed in subsection 6.4.

### 5.1 Experimental procedure

**Fig. 5:**
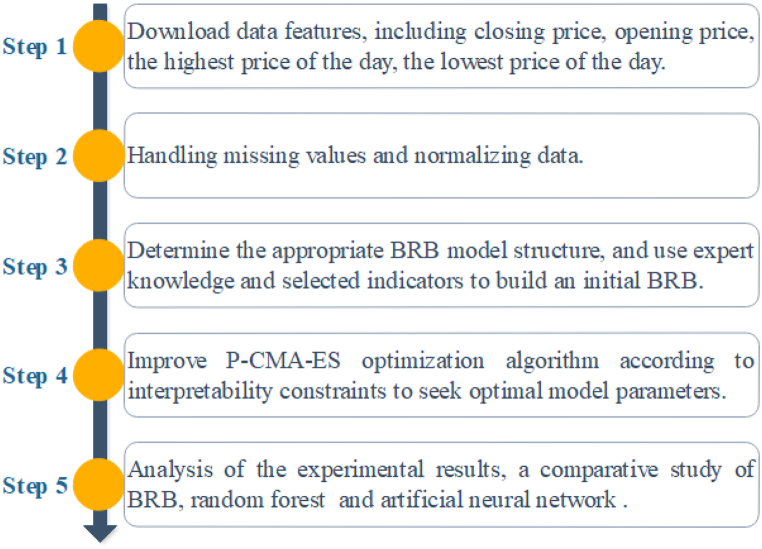
The experimental process of stock price prediction.

The experiment mainly consists of the parts of data downloading, data processing, initial model building, model training, and comparison and analysis of results. The experimental process of stock price prediction is shown in Fig. 5.

### 5.2 Dataset and experimental setup

The changes in stock data are nonsmooth, nonnormal, and nonlinear, and the constructed stock price prediction system not only can handle uncertain information but also the model must be interpretable and the results must be trustworthy [4].

**Fig. 6:**
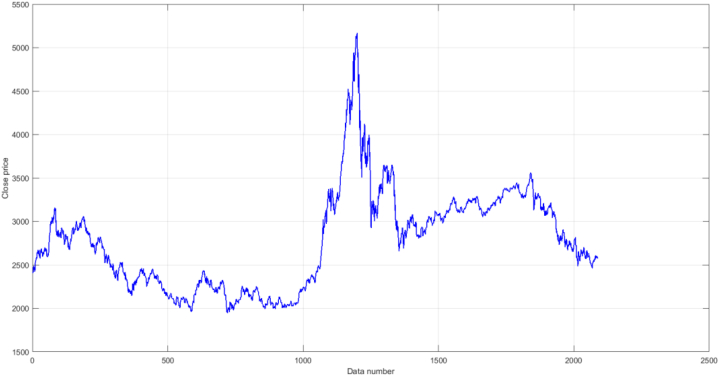
Overall movement of the SSE Index.

The Shanghai Stock Composite Index (SSE) is selected for the experiments to evaluate the study. Based on existing studies [52,53], the metrics of the initial historical data were selected as the attributes of the model. Stock trading indicators covering the period from July 6, 2010, to January 31, 2019, when the stock market was closed on Saturday and Sunday, and the final dataset included 2087 data points of the index. In this paper, the first 80% of the dataset is the training data, and the last 20% is the test data. The overall movement of the SSE index is shown in Fig. 6. The development environment for the experiment is Windows 10 and 8th Gen Intel (R) Core (TM) i5-8265U. The experiment was conducted in Matlab without using other libraries.

Because of the different units of the data factors, the gradient of the model training will be slowed down due to the different magnitudes of the data, so it is necessary to standardize the data and control the data range to (0,1). The standardization formula is shown in Eq. (43):

(43) $$\tilde{x}=\frac{x-x_{\min}}{x_{\max}-x_{\min}}$$

The issue of missing data arises due to sampling loss and statistical incompleteness, making it difficult to analyze the data. Different samples have varying degrees of data corruption, which requires a customized approach for each case. In instances where most of the historical data is missing, the direct elimination method is used to exclude the data. On the other hand, the mean-filling method is used to fill in missing data for factors with only a few missing historical data, ensuring the integrity of the data.

**Fig. 7:**
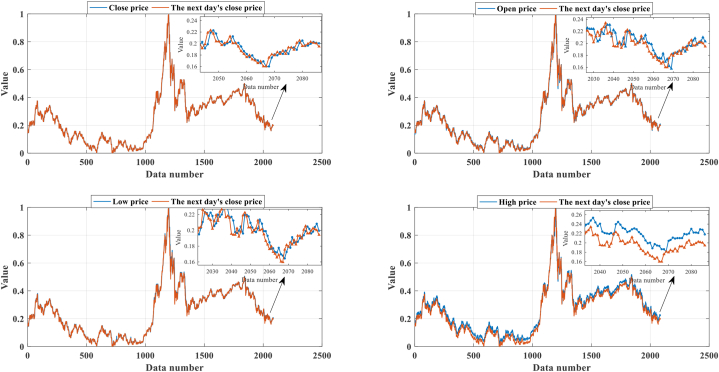
The four attributes after normalization.

**Fig. 8:**
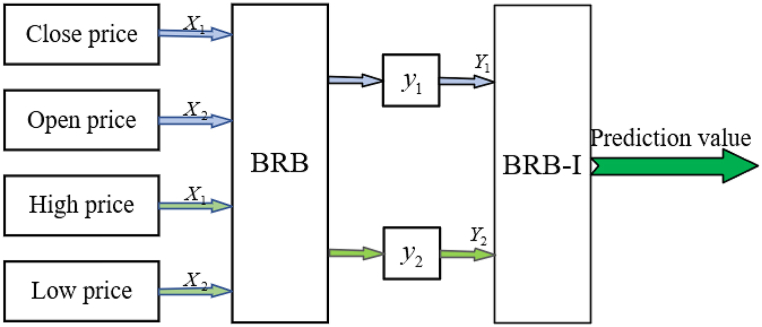
The structure of the HBRB-I for stock prediction.

Then, the relationship between the four normalized attributes and the next day's stock price is shown in Fig. 7. Fig. 8 shows the structure of the HBRB-I, where the first two BRBs use the initial expert knowledge base for prediction direction adjustment, and the latter performs interpretable optimization to achieve accurate prediction. Expert has many years of experience in the field and has a good reputation, so in this paper, expert knowledge is considered reliable.

According to the judgment of expert knowledge, five semantic values are selected to describe the system state, namely, “very low” (VL), “low” (L), “medium” (M), “high” (H) and “very high” (VH). The weights of the attributes and the initial reference values of BRB1, BRB2, and BRB-I are shown in Table 1. The initial rules and the initial rule weights are shown in Table 2. Experts believe that the attributes and rules of this experiment are important. The expert gave a range but did not give a specific value. Therefore, this article is temporarily set to all 1 for optimization in subsequent training.

**Table 1:** The initial weight of attributes and reference values.

Attribute (*X*)	Attribute weight	Attribute weight constraint	Referential values (*A)*				
	$\delta_{i}$	$\delta_{i}∼C_{1}$	VL	L	M	H	VH
Close price	1	0.6–1	0	0.2	0.4	0.7	1
Open price	1	0.6–1	0	0.2	0.4	0.7	1
High price	1	0.6–1	0	0.2	0.4	0.7	1
Low price	1	0.6–1	0	0.2	0.4	0.7	1
y_1_	1	0.6–1	0	0.2	0.4	0.7	1
y_2_	1	0.6–1	0	0.2	0.4	0.7	1
Output(β*)*			0	0.2	0.4	0.7	1

**Table 2:** The initial rules of the three BRBs.

$X_{1}\wedge X_{2}$	The rule weight constraint	Rule weight	The initial belief	The belief constraint
	$\theta_{k}∼C_{1}$	$\theta_{k}$	$\left\{\beta_{1}^{k},\beta_{2}^{k},\beta_{3}^{k},\beta_{4}^{k},\beta_{5}^{k}\right\}$	$\left\{\beta_{1}^{k},\beta_{2}^{k},\beta_{3}^{k},\beta_{4}^{k},\beta_{5}^{k}\right\}$
VL ∧ VL	0.5–1	1	{0.95,0.05,0,0,0}	{0.9–1,0–0.1,0–0.1, 0–0.1,0–0.1}
VL ∧ L	0.5–1	1	{0.6,0.4,0,0,0}	{0.5–0.7,0.3–0.5,0–0.1, 0–0.1,0–0.1}
VL ∧ M	0.5–1	1	{0.3,0.5,0.2,0,0}	{0.2–0.4,0.4–0.6,0.1–0.3, 0–0.1,0–0.1}
VL ∧ H	0.5–1	1	{0.1,0.4,0.4,0.1,0}	{0–0.2,0.3–0.5,0.3–0.5, 0–0.2,0–0.1}
VL ∧ VH	0.5–1	1	{0.1,0.2,0.4,0.2,0.1}	{0–0.2,0.1–0.3,0.3–0.5, 0.1–0.3,0–0.2}
L ∧ VL	0.5–1	1	{0.6,0.4,0,0,0}	{0.5–0.7,0.3–0.5,0–0.1, 0–0.1,0–0.1}
L ∧ L	0.5–1	1	{0.2,0.7,0.1,0,0}	{0.1–0.3,0.6–0.8,0–0.2, 0–0.1,0–0.1}
L ∧ M	0.5–1	1	{0.1,0.4,0.4,0.1,0}	{0–0.2,0.3–0.5,0.3–0.5, 0–0.2,0–0.1}
L ∧ H	0.5–1	1	{0,0.25,0.5,0.25,0}	{0–0.1,0.2–0.3,0.4–0.6, 0.2–0.3,0–0.1}
L ∧ VH	0.5–1	1	{0,0.1,0.3,0.5,0.1}	{0–0.1,0–0.2,0.2–0.4, 0.4–0.6,0–0.2}
M ∧ VL	0.5–1	1	{0.3,0.5,0.2,0,0}	{0.2–0.4,0.4–0.6,0.1–0.3, 0–0.1,0–0.1}
M ∧ L	0.5–1	1	{0.1,0.4,0.4,0.1,0}	{0–0.2,0.3–0.5,0.3–0.5, 0–0.2,0–0.1}
M ∧ M	0.5–1	1	{0,0,1,0,0}	{0–0.1,0–0.1,0.9–1, 0–0.1,0–0.1}
M ∧ H	0.5–1	1	{0,0.1,0.4,0.4,0.1}	{0–0.1,0–0.2,0.3–0.5, 0.3–0.5,0–0.2}
M ∧ VH	0.5–1	1	{0,0,0.2,0.5,0.3}	{0–0.1,0–0.1,0.1–0.3, 0.4–0.6,0.2–0.4}
H ∧ VL	0.5–1	1	{0.1,0.5,0.3,0.1,0}	{0–0.2,0.4–0.6,0.2–0.4, 0–0.2,0–0.1}
H ∧ L	0.5–1	1	{0,0.25,0.5,0.25,0}	{0–0.1,0.2–0.3,0.4–0.6, 0.2–0.3,0–0.1}
H ∧ M	0.5–1	1	{0,0.1,0.4,0.4,0.1}	{0–0.1,0–0.2,0.3–0.5, 0.3–0.5,0–0.2}
H ∧ H	0.5–1	1	{0,0,0.1,0.7,0.2}	{0–0.1,0–0.1,0–0.2, 0.6–0.8,0.1–0.3}
H ∧ VH	0.5–1	1	{0,0,0,0.4,0.6}	{0–0.1,0–0.1,0–0.1, 0.3–0.5,0.5–0.7}
VH ∧ VL	0.5–1	1	{0.1,0.2,0.4,0.2,0.1}	{0–0.2,0.1–0.3,0.3–0.5, 0.1–0.3,0–0.2}
VH ∧ L	0.5–1	1	{0,0.1,0.4,0.4,0.1}	{0–0.1,0–0.2,0.3–0.5, 0.3–0.5,0–0.2}
VH ∧ M	0.5–1	1	{0,0,0.2,0.5,0.3}	{0–0.1,0–0.1,0.1–0.3, 0.4–0.6,0.2–0.4}
VH ∧ H	0.5–1	1	{0,0,0,0.4,0.6}	{0–0.1,0–0.1,0–0.1, 0.3–0.5,0.5–0.7}
VH ∧ VH	0.5–1	1	{0,0,0,0.05,0.95}	{0–0.1,0–0.1,0–0.1, 0–0.1,0.9–1}

Based on the above settings, the HBRB-I model for stock price movement prediction is developed as Eq. (44):

(44) $$\begin{array}{c} \text{IF}\left(X_{1}\;is\;A_{1}\right)\Lambda \left(X_{2}\;is\;A_{2}\right)\text{,}\\ \text{THEN}\left\{\left(VL,\beta_{1}\right),\left(L,\beta_{2}\right),\left(M,\beta_{3}\right),\left(H,\beta_{4}\right),\left(VH,\beta_{5}\right)\right\}\\ \text{with}\;a\;\text{rule}\;\text{weight}\;\theta_{k}=1\;\left(k=1,2,\ldots ,25\right)\\ \text{and}\;\text{attribute}\;\text{weights}\;\delta_{i}=1\;\left(i=1,2\right)\text{,} \end{array}$$

### 5.3 Experimental procedure of the model

**Fig. 9:**
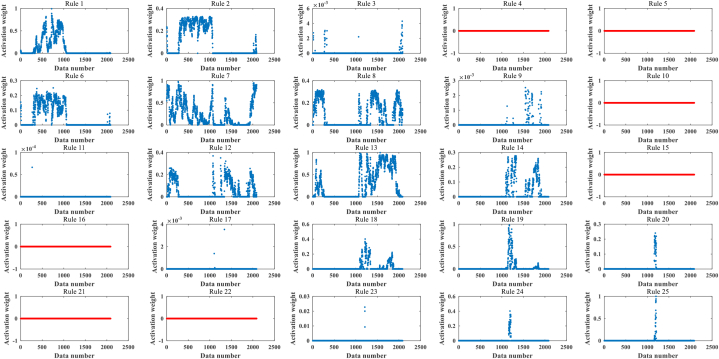
Analysis of HBRB-I rule activation weights.

First, the dataset is analyzed for activation weights, and the inactivation weights are flagged. Fig. 9 shows the activation weights for each rule of HBRB-I.

The figure shows that rules 4, 5, 10, 15, 16, 21, and 22 are not activated, the activation status is shown as Eq. (45):

(45) $$\omega =\left\{\omega_{1},\omega_{2},...,\omega_{k}\right\}=\left\{1,1,1,0,0,1,1,1,1,0,1,1,1,1,0,0,1,1,1,1,0,0,1,1,1\right\}\text{.}$$

This indicates that in a limited dataset, these rules do not have an impact on the results. Therefore, these rules are marked down and corrected during the optimization process if the relevant parameters are adjusted to avoid destroying the interpretability of the model.

The initial reference value and the feasible interval of the best reference value are given in Table 1, Table 2 The optimized rules in Table 3 show the effectiveness of the adjusted optimization algorithm.

**Table 3:** The optimized rules.

No.	$X_{1}\wedge X_{2}$	Rule weight	The optimized belief
	$y_{1}\wedge y_{2}$	$\theta_{k}$	$\left\{\beta_{1}^{k},\beta_{2}^{k},\beta_{3}^{k},\beta_{4}^{k},\beta_{5}^{k}\right\}$
1	VL ∧ VL	0.554	{0.9,0.1,0,0,0}
2	VL ∧ L	0.602	{0.55,0.39,0.02,0.02,0.02}
3	VL ∧ M	0.925	{0.25,0.48,0.18,0.05,0.04}
4	VL ∧ H	1	{0.1,0.4,0.4,0.1,0}
5	VL ∧ VH	1	{0.1,0.2,0.4,0.2,0.1}
6	L ∧ VL	0.988	{0.62,0.38,0,0,0}
7	L ∧ L	0.701	{0.25,0.72,0.02,0.01,0}
8	L ∧ M	0.934	{0.16,0.36,0.36,0.06,0.06}
9	L ∧ H	0.817	{0,0.21,0.55,0.24,0}
10	L ∧ VH	1	{0,0.1,0.3,0.5,0.1}
11	M ∧ VL	0.975	{0.28,0.52,0.2,0,0}
12	M ∧ L	0.812	{0.01,0.43,0.43,0.1,0.03}
13	M ∧ M	0.518	{0.01,0.01,0.96,0.01,0.1}
14	M ∧ H	0.968	{0.01,0.14,0.33,0.46,0.06}
15	M ∧ VH	1	{0,0,0.2,0.5,0.3}
16	H ∧ VL	1	{0.1,0.5,0.3,0.1,0}
17	H ∧ L	0.808	{0,0.32,0.48,0.2,0}
18	H ∧ M	0.904	{0.01,0.06,0.39,0.42,0.12}
19	H ∧ H	0.858	{0,0.05,0.05,0.75,0.15}
20	H ∧ VH	0.778	{0,0,0.04,0.43,0.53}
21	VH ∧ VL	1	{0.1,0.2,0.4,0.2,0.1}
22	VH ∧ L	1	{0,0.1,0.4,0.4,0.1}
23	VH ∧ M	0.587	{0,0.02,0.26,0.4,0.32}
24	VH ∧ H	0.828	{0,0,0,0.37,0.63}
25	VH ∧ VH	0.785	{0.01,0.01,0.01,0.07,0.9}

**Fig. 10:**
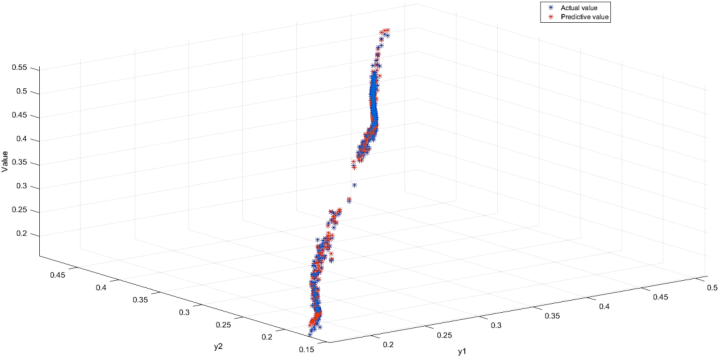
Model prediction results.

The parameters of the optimization algorithm are given in Table 4. The prediction results are shown in Fig. 10. The average MSE of experimental results is 1.69E-04, which indicates the accuracy of the method. To demonstrate the robustness, 20 replicate experiments were performed. The variance in MSE (1.42E-05) was much smaller than the mean MSE (1.79E-04).

**Table 4:** Parameters of the optimization algorithm.

The parameter	Initial set value
Generation G	200
Population number λ	25
Step length $\varepsilon^{0}$	0.2
The Euclidean distance d	2.4

### 5.4 Analysis of experimental results

To better evaluate the model performance, two currently popular prediction models, Random Forest (RF) and Artificial Neural Network (ANN) were selected for comparison experiments. Additionally, the BRB built from expert knowledge (BRB0), the original BRB (BRB1), and the HBRB-I are compared. In this paper, the mean squared error (MSE) is selected for comprehensive evaluation as well as a comparison of models, which can be calculated as Eq. (46):

(46) $$MSE=\frac{1}{n}\sum_{i=1}^{n}{\left(y_{i}-{\hat{y}}_{i}\right)}^{2}$$

where $y_{i}$ is the actual value, ${\hat{y}}_{i}$ is the predicted value, and n is the data number.

#### 5.4.1 Accuracy analysis

In the accuracy analysis, the experimental results are described in two parts. To effectively conduct the comprehensive evaluation of the model, Mean Absolute Error (MAE) is added as an evaluation indicator in this section. MAE is the mean of absolute error, and the smaller the value, the better the prediction effect. The calculation formula is shown in Eq. (47). BRB0, BRB1, and BRB2, with RF and ANN, and the results values are given in Table 5.

(47) $$MAE=\frac{1}{n}\sum_{i=1}^{n}\left|y_{i}-{\hat{y}}_{i}\right|$$

**Table 5:** Comparison of BRB0, BRB1, BRB2, RF and ANN model predictions.

Part	Models	MSE	MAE
Part I	BRB0	8.68E-04	0.025
	BRB1	1.15E-04	0.009
	BRB2	1.69E-04	0.011
Part II			
	RF	3.10E-04	0.014
	ANN	**9.61**E**-05**	**0.007**

**Fig. 11:**
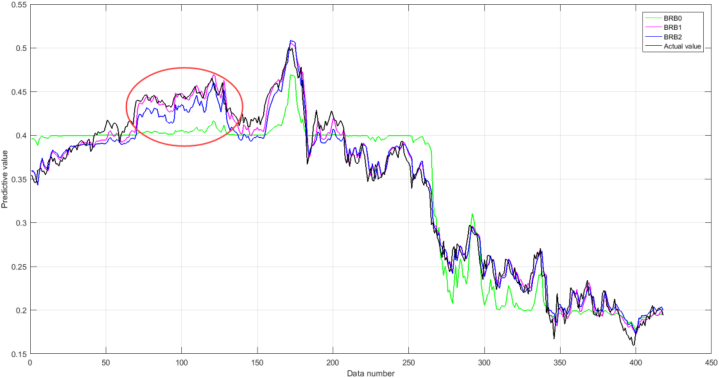
The compare results of three BRBs.

In the first part, the outputs and actual values of BRBs are shown in Fig. 11. As shown in the flagged line in the figure: the accuracy of BRB2 is poor compared to BRB1, and the deviation of BRB0 from the actual value is largely due to the limitation of the initial expert knowledge, which also leads to the reduction of the accuracy of the interpretable BRB2 considering the expert knowledge. Therefore, experts can analyze the initial model mechanism and slightly adjust the knowledge. It can be concluded that the constructed HBRB-I model constructs the model through the model mechanism and optimizes the model by using data samples, which not only guarantees the accuracy of the model but also has a certain degree of interpretability in the modeling process.

**Fig. 12:**
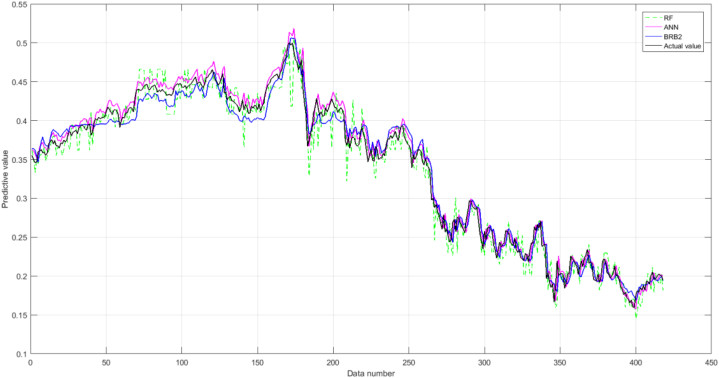
The results generated by RF, ANN, and BRB2.

The second part is the comparison of BRB2, RF, and ANN. From Fig. 12, it can be seen that, as a whole, ANN's performance is significantly better than RF's and slightly better than BRB2's. The reason is that the data-driven model does not consider the model mechanism and seeks to obtain higher accuracy. However, the BRB2 significantly outperformed the RF in terms of performance and the BRB2 model has outstanding interpretability advantages, and ANN cannot clearly describe uncertainty and give the results of outcome distribution evaluation.

#### 5.4.2 Interpretability analysis

##### 5.4.2.1 Analysis of distinguishability of reference values

**Fig. 13:**
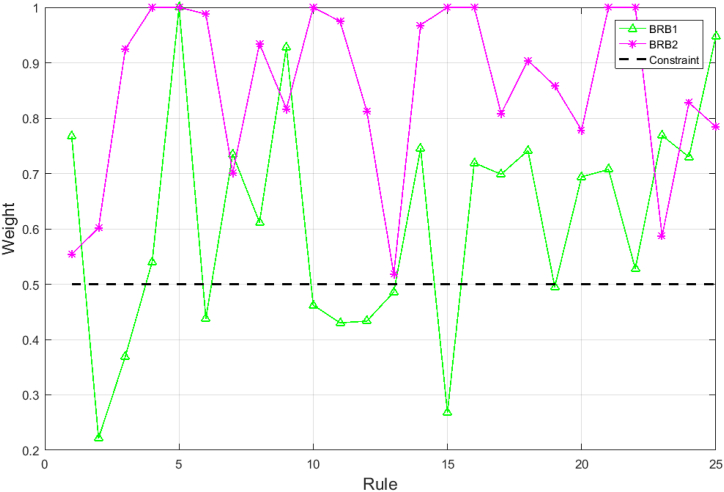
Comparison of rule weights of BRB1 and BRB2.

In Fig. 13, the rule weights of BRB2 satisfy the corresponding criteria, while BRB1 is mostly far from the initial expert knowledge or exceeds the constraint. Compared to other data-driven models, the parameter settings of BRB2 are meaningful, and the inference process is transparent. In this paper, the attribute weights of y_1_ and y_2_ are 0.648 and 0.636, respectively. This also shows that these attributes are almost equally important for stock price prediction.

##### 5.4.2.2 Interpretability analysis with unactivated rules removed

**Fig. 14:**
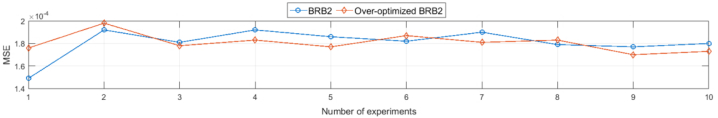
Comparison of the MSEs of BRB2 and over-optimized BRB2.

For interpretability criterion 10, ten experiments of parameter over-optimization were performed, and the unconstrained optimization of the unactivated rules was disregarded by the optimization algorithm. The experimental results of the over-optimized BRB2 are compared with the results of the constrained BRB2, as shown in Fig. 14. The optimization for removing the inactivated parameters neither affects the accuracy nor affects the interpretability. The relevant parameters of the inactivated rules are adjusted in over-optimized BRB2, making the optimized parameters uninterpretable and reducing the trust of researchers. Therefore, it is necessary to protect the interpretability of rules.

##### 5.4.2.3 Reasonable belief distribution analysis

**Fig. 15:**
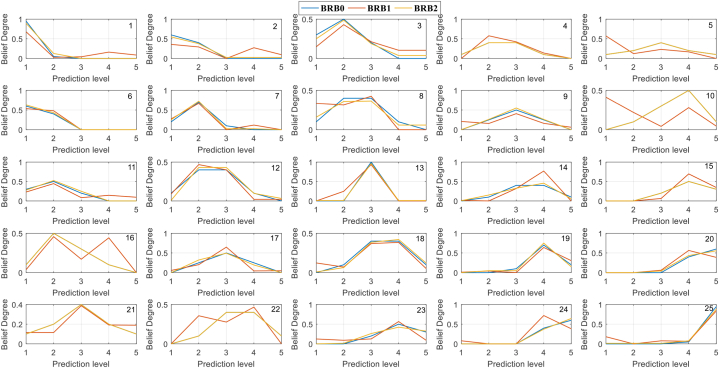
The belief distributions of each rule.

Fig. 15 shows the belief distributions of BRBs. BRB2 and BRB0 have similar belief distributions. In addition, the corresponding belief distributions of the unactivated rules are not mobilized, which fully guarantees interpretability and increases the expert's trust in the model. In contrast, most of the rules of BRB1 seriously deviate from the initial expert knowledge, and even some rules have obvious problems, which are difficult to interpret. For example, the seventh rule is shown in Fig. 15. It further shows that the fine-tuning of the optimization algorithm is very important for interpretability.

##### 5.4.2.4 Belief degree analysis of results

**Fig. 16:**
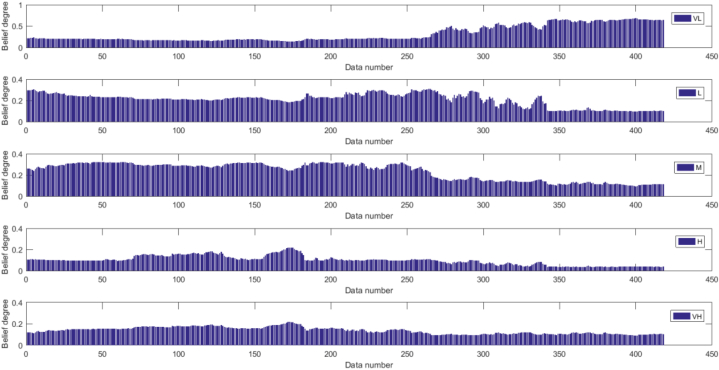
The results distribution of BRB1.

**Fig. 17:**
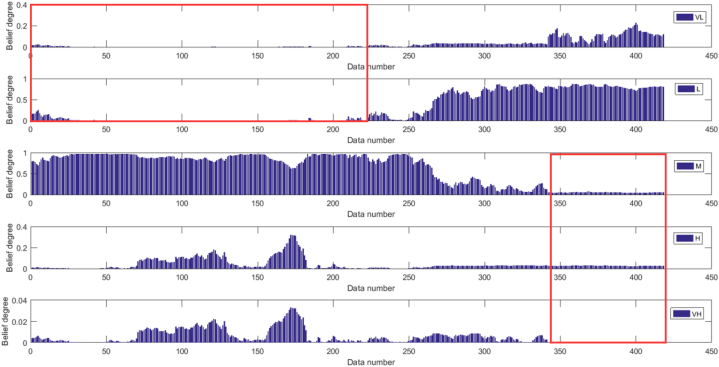
The results distribution of BRB2.

Fig. 16, Fig. 17 depict the results of the BRB1 and BRB2 distributions, showing the transparency of the BRB. Both BRB1 and BRB2 can describe the results of the experiment with clear semantics However, the red boxed line in Fig. 17 shows that BRB2 is more capable of handling uncertainty in real systems. In the final prediction results compared to BRB1, BRB2 not only maintains high accuracy but it can be seen that the belief level of each resultant reference point keeps increasing, while the belief level relative to other reference points keeps decreasing, which is understandable to the researcher.

#### 5.4.3 Robustness analysis

**Fig. 18:**
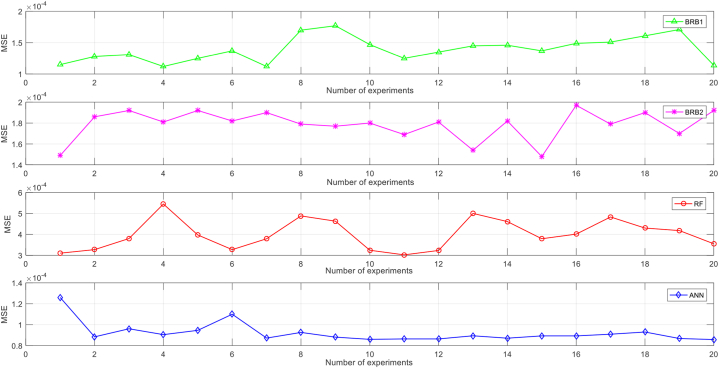
MSE comparison of four models.

The optimization process was repeated 20 times for each of the BRB1, BRB2, RF, and ANN models to verify the robustness of the method. Table 6 shows the robustness results of the experiment and the comparison of the MSEs of the prediction results for BRB1, BRB2, RF, and ANN models are shown in Fig. 18.

**Table 6:** The robustness of the optimization algorithm.

MSE	BRB1	BRB2	RF	ANN
Max	1.77E-04	1.97E-04	5.45E-04	1.26E-04
Min	1.12E-04	1.48E-04	3.02E-04	8.58E-05
Average	1.39E-04	1.79E-04	3.99E-04	9.22E-05
The standard deviation of MSE	1.99E-05	1.42E-05	7.18E-05	9.67E-06

It can be seen that the robustness of BRB2 is better than BRB1. The reason is that the optimization algorithm considering the interpretability criterion limits the optimization search space, the search fluctuation is small, and the optimized parameters are similar to the expert knowledge.

In the literature [54], it is pointed out that the lack of interpretability is a major challenge for artificial neural networks, especially in applications where incorrect or biased predictions can have significant consequences. Although the article provides a detailed survey of different approaches to improve ANN interpretability, it does not reach a clear consensus on which approach or combination of approaches is most effective or appropriate for different types of applications or datasets. The article also acknowledges that many of the existing methods for improving ANN interpretability are still relatively emerging and may require further development and validation before they can be widely adopted.

#### 5.4.4 Convergence analysis

**Fig. 19:**
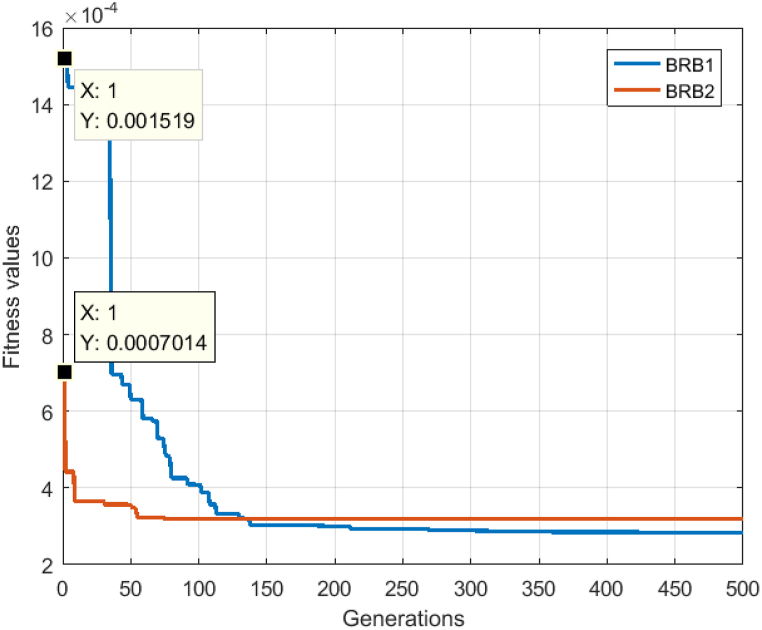
The fitness values of the BRB1 and BRB2 models over 500 generations.

Fig. 19 shows the convergence speed of the BRB1 and BRB2 models. At the beginning of the optimization, the convergence speed of BRB1 is slower, while that of BRB2 is faster than that of BRB1. The reason is that the initial parameters of the BRB2 model refer to a lot of expert knowledge, and due to the limitation of interpretable criteria, the optimized solution can find the best solution faster. However, it can also be seen that the optimization accuracy of BRB1 is gradually better than that of BRB2 in the end, which is due to the restriction of the parameter optimization search range of BRB2, but this is the result of damaging the interpretability, and the accuracy gap between the two models is not too large.

#### 5.4.5 Cross-validation analysis

Cross-validation is important because it helps to evaluate the performance of the model on new data [55]. By splitting the data into training and validation sets multiple times, cross-validation can provide a more accurate estimate of the model's generalization performance and can help prevent overfitting of the training data. In this paper, all samples are first divided into 5 groups, where each group is 20% of the data set. Then, one group at a time is used as the test set and the remaining four groups as the training set, and finally, the results of the five modeling runs are evaluated together. The experimental results are shown in Table 7.

**Table 7:** Model accuracy under cross-checking.

Models	Round 1	Round 2	Round 3	Round 4	Round 5	Average
BRB0	8.68E-04	6.76E-04	6.22E-04	8.07E-04	1.47E-03	8.89E-04
BRB1	1.50E-04	1.71E-04	8.82E-05	3.95E-04	4.06E-04	2.42E-04
BRB2	1.69E-04	2.12E-04	9.59E-05	6.94E-04	5.36E-04	3.41E-04

#### 5.4.6 Applicability analysis

To verify the applicability of the proposed method, the Shanghai Stock Composite Index (SSE) and the Dow Jones Industrial Average (DJI) from 2019/1/31–2022/6/17 are chosen in this paper. The relevant data comes from the public dataset of the Kaggle database: https://www.kaggle.com/datasets/gelasiusgalvindy/stock-indices-around-the-world. The experimental results are shown in the following Table 8. The experimental results show that the proposed method is applicable in stock forecasting.

**Table 8:** Accuracy testing of the latest SSE and DJI datasets collected.

Models	The Shanghai Stock Composite Index	The Dow Jones Industrial Average Index
BRB0	2.3E-03	1.2E-03
BRB1	**1.1E-03**	5.35E-04
BRB2	1.2E-03	6.81E-04
RF	2.7E-03	8.17E-04
ANN	1.2E-03	**4.61E-04**

## 6 Conclusion

In this paper, we construct the HBRB-I model based on the BRB to provide a highly accurate and trustworthy method for predicting stock price movements. Researchers have demonstrated the ability of the BRB expert system to predict future stock prices, and in this research paper, the HBRB-I model enhances the structural scalability as well as interpretability of the BRB, showing the advantages of the HBRB-I model in the stock market. First, the initial historical data of the stock with expert knowledge is used as the model inference machine by the evidence inference method, and then the parameters are optimized to reduce the MSE. The final result indicates that the HBRB-I model achieves an accuracy of 1.69E-04 while improving the interpretability, which basically reaches the accuracy of the initial BRB. Among them, the interpretability of BRB is fully considered, and several criteria are constructed. In addition, the process of constructing the overall interpretability is given.

The HBRB-I model has proven to be efficient in dealing with the stock price movement prediction problem. However, the rule base in this paper is built on nearly a decade of collected data to make forecasts for the short-term future and does not take into account sudden trend changes, which has limitations. We plan to continue our research in the following aspects in the future:

a) Designing an interpretable BRB that can dynamically add or remove rules, on the one hand, streamline the size of the rule base and enhance readability. On the other hand, it can make the rule base satisfy its integrity in case of system inputs that exceed the knowledge experience.

b) ER methods have been widely used as inference engines for BRB, but it is important to develop an inference algorithm for approximate causal inference considering more interpretable BRB models, which will be the focus of the next research.

c) The study demonstrated the validity of stock price forecasting using the four characteristics, proving that they are indeed reliable variables for such forecasting. However, it is important to continue researching and reviewing other potential variables and characteristics to obtain more accurate and reliable stock price predictions.

## Author contribution statement

Xiuxian Yin: Conceived and designed the experiments; Wrote the paper.

Hongyu Li: Performed the experiments.

Xin Zhang: Conceived and designed the experiments.

Yujia Chen: Analyzed and interpreted the data.

Wei He: Contributed reagents, materials, analysis tools or data.

## Data availability statement

The authors do not have permission to share data.

## Declaration of competing interest

The authors declare that they have no known competing financial interests or personal relationships that could have appeared to influence the work reported in this paper.
